# Treatment of Type Two Slap Lesion With Anatomic Suture Anchor Repair Without Biceps Tenotomy Or Tenodesis

**DOI:** 10.2174/1874325001812010324

**Published:** 2018-07-31

**Authors:** Chadwick C. Prodromos, Susan Finkle, Alexander Dawes, Ji Young Baik

**Affiliations:** The Illinois Orthopaedic Foundation, Orthopaedic Surgery Rush University (Ret), Chicago, C.A, United States

**Keywords:** Shoulder, SLAP lesion, SLAP repair, Biceps tenotomy, Biceps tenodesis, Surgery

## Abstract

**Background:**

Poor results after repair of type 2 SLAP tears are relatively common and some have reported better results after biceps tenodesis or tenotomy than repair. In addition, some believe that the long head of the biceps is expendable. Therefore, many now favor biceps tenotomy or tenodesis over biceps anchor repair either in all patients or in older patients, reserving SLAP lesion repair only for young athletes.

**Hypothesis:**

We hypothesized that repair of the biceps anchor of the labrum would be effective in all patients regardless of age provided that care was taken not to overtighten the labrum and that rotator cuff pain as the primary pain generator had been ruled out.

**Methods:**

All patients with type 2 SLAP lesion repair by the senior author since he began repairing them with suture anchors were prospectively evaluated. Patients with more than one other concomitant procedure, simultaneous rotator cuff repair or worker’s compensation status were excluded.

**Results:**

77% of patients were available for minimum two year followup. No patient had subsequent surgery or manipulation under anesthesis as a result of their SLAP repair. Standardized shoulder test score increased by 4 points. Mean SANE score decreased from 53 pre-op to 14 post-op. Results were the same in those over versus under 40 years of age.

**Conclusion:**

Anatomic repair of Type 2 SLAP lesions at the biceps anchor without biceps tenodesis or tenotomy can produce good results in patients of all ages.

## 1 INTRODUCTION

The glenoid labrum was originally thought to be composed of fibrous tissue until it was shown to be fibrocartilaginous by Prodromos *et al*. [1] indicating that it is at least partially loaded in compression as well as tension. The treatment of type 2 labral tears with detachment of the biceps anchor (SLAP lesions), as defined by Stephen Snyder [2] is controversial. Because reported unsatisfactory results after surgical treatment are relatively common [3-5], it has become customary for surgeons to perform biceps tenodesis or tenotomy instead of labral repair, particularly in patients over forty years of age [5-9]. While some studies have seemed to indicate that the long head of the biceps is “expendable” [10], we are not convinced. Unless there is no other effective treatment alternative, we feel that it is inherently undesirable to sacrifice a healthy tendon in the interests of treating another problem: and – absent neuromuscular disorder – no other tendon is sacrificed in this manner. While the literature would seem to indicate near universal safety after biceps tenodesis/tenotomy [3, 6-8, 11], we not infrequently see patients who have problems, such as biceps muscle cramping, a “popeye” biceps or tenodesis incisional pain, that seem directly attributable to the prior performance of these procedures.

We also believe that many of the cases of failed SLAP repair occur because the SLAP lesion was actually not the cause of the patient’s pain. Most commonly in our opinion, anterolateral rotator cuff pain is the confounding entity. In cases where anterolateral pain exists we perform an “impingement test”, injecting the subacromial bursa with lidocaine [12]. If the patient’s pain is substantially relieved, we generally do not proceed with SLAP repair, even if an MRI arthrogram shows an apparently clear type 2 SLAP lesion. We do not believe that all such tears require surgery to achieve a satisfactory result. We also require a history of traction or compression trauma to the shoulder [13] and the presence of posterior pain, in most cases, for surgery to be indicated.

One of the adverse events after SLAP repair that most drives the trend to sacrifice the biceps is stiffness [4, 5, 7, 14, 15]. However, we feel that if the superior labrum and biceps, I.E. the biceps anchor, are anatomically restored without tightening, there should not be any reason why stiffness would occur. We thus believe that most cases of pain and stiffness after type 2 SLAP repair likely occur because the labrum was not detached at the biceps anchor to begin with, such that tacking it down cannot help but overtighten the biceps; or if the biceps anchor was indeed initially detached that it was overtightened during repair.

We thus hypothesized that if care were taken preoperatively to make sure that the SLAP lesion was the cause of the patient’s pain, and if a surgical repair with sutures on the external superior surface of the labrum that does not tether the free central border of the labrum and that carefully avoids tightening the labrum or biceps were used: then the Type 2 SLAP repair would not result in stiffness or worsening. We further hypothesized that if conservative treatment of a bona fide type 2 tear had failed then repair of the biceps anchor, rather than biceps tenodesis or tenotomy, would be similarly effective in all patients regardless of age: with no worsening in outcome in patients over 40 compared to those under 40. The purpose of this study was to test these hypotheses.

## 2 METHODS

We prospectively collected data on all patients who had Type 2 SLAP lesion repairs by the senior author from March 2005 when he began repairing them with suture anchors instead of tacks, to December 2015. Patients were included who had arthroscopic SLAP repair but no rotator cuff surgery, either repair or debridement, to avoid confounding rotator cuff treatment results, especially stiffness, with SLAP repair results. Included patients had SLAP repair in isolation, or with only one other non rotator cuff surgical procedure, the results from which would be unlikely to be confused with the results of SLAP repair. There were 72 patients who had SLAP repair and no rotator cuff pathology. From this group all patients who had SLAP repair in isolation, or with only 1 additional procedure were selected: A total of 44 patients. Since multiple studies have shown worse outcomes in patients with workers compensation [9, 16, 17] possibly due to non medical reasons, we eliminated all worker’s compensation patients, and patients with personal injury where payment of medical bills was made by a third party. All payment was by commercial or government insurance.

This left 30 shoulders (28 patients) with type 2 SLAP lesions either in isolation or in combination with one other procedure not related to the rotator cuff. The breakdown among them was: 20 patients with isolated SLAP repair, four with SLAP repair and distal clavicectomy, three with SLAP repair and arthroscopic Bankart repair, two with SLAP repair and microfracture (for grade 4 small glenoid defects), and one with SLAP repair and loose body removal. Age range was 17 to 55 years with a median of 31 (standard deviation = 14.9 years). There were 25 males and 5 females. Patients were asked to complete a SANE score for pain and a Simple Shoulder Test assessment prior to surgery and again at 2 year+ followup. Patient satisfaction was assessed by asking if the patient would choose to have the surgery again.

## 3 SURGICAL TECHNIQUE

 General anesthetic with pre-operative oral gabapentin, but without a nerve block, was used in all cases. Bio-absorbable suture anchors with two high strength sutures per anchor were used in all cases after debridement of the superior surface of the bony glenoid. Mattress sutures were used with knots tied external to the labrum. The labrum and biceps were not tightened beyond their estimated normal anatomic tension. The biceps anchor was not considered torn at surgery, and a repair was not performed, unless the biceps anchor could be completely separated superiorly from the superior bony glenoid with no tissue fibers seen attaching the biceps anchor to the bony glenoid. We typically place a single arthroscopic absorbable double loaded anchor at the superior pole of the glenoid and then place a mattress sutures anteriorly and posteriorly. In large tears we place a second anchor posteriorly to the first.

## 4 POSTOPERATIVE REHABILITATION

 A shoulder immobilizer without an elevating pillow was applied at surgery. The patient was urged to discard it completely as soon as possible, and in all cases by the end of the first post-operative week. Post-operative analgesia was achieved with topical ice and hydrocodone/acetaminophen. Rehabilitation consisting of home external rotation exercise was begun one week post-operatively. Active elevation of the elbow, *i.e*. shoulder forward flexion, abduction or extension, was not allowed until postoperative week six although free internal and external rotation of the shoulder and free use of the elbow, wrist and hand were allowed immediately post-operatively. Free Active Range of Motion (AROM) of the shoulder was allowed at 6 weeks post-operatively, but without lifting heavy objects. Physical therapy, consisting of passive ROM stretching and AROM with strengthening, was begun at post-operative week 10, with active elevation limited to 40 degrees in all planes to avoid rotator cuff irritation. Physical therapy was scheduled twice weekly and in most cases was discontinued before the 6^th^ post-operative month when patients were generally discharged with unrestricted activity.

## 5 RESULTS

Twenty-four of 30 patients (80%) had minimum 2 year followup (see Table **1**) (mean 57.5 months (range 24 to 101 months). SST scores were obtained on all 24. Seventeen patients had an improved SST at time of final followup. One had no change. Five patients had no recorded pre-op SST. Of those five, three had a perfect final SST of 12, two had a score of 11, indicating likely improvement in all of these patients. One patient had a decreased SST from pre-op to final follow-up at 24 months post-op from 4 to 2, although he had a good result when he was released 4 months postoperatively (improved range of motion, FF to 170, good strength, and decreased pain; and stated at long term follow-up that he would elect to have the procedure again). Five patients (6 shoulders) were lost to follow-up after six months or less follow-up. All had a satisfactory result at that visit and were discharged (except one who could not continue to follow up after 2 months due to a diagnosis of cancer for whom it was too soon to evaluate outcome). One of these 5 patients had follow-up at 7 years with perfect function but no recorded SST. The mean and median SST change was a four point increase for the group.

Pre and post op SANE scores were obtained on 18 patients, with a mean improvement of 36.7 at final follow-up. Mean preop SANE was 52.5. Postop SANE scores were obtained on 24 patients with a mean of 13.5. There was a significant difference seen between pre and post op scores for both the SST and the SANE.

Twenty-two patients responded to the satisfaction question, with 21 of them saying they definitely or probably would choose to have the surgery again. One patient stated they probably would not choose the surgery again, despite having a post op SST of 12 and a SANE of 0.

Patients were separated into two groups by age, one group 39 years of age and under, and one 40 and over. The breakdown of these 2 groups can be seen in Table **2**. There were no differences found in the functional outcomes of the 2 groups. The SST was not significantly different between the two age groups before or after surgery. The SANE score was not significantly different after surgery, but was before surgery, with the 40 and over group presenting with significantly higher SANE scores before surgery (p=0.01).

There were no surgical complications. No patient developed permanent stiffness or required manipulation under anesthesia. No patient had subsequent surgery related to their original repair. However, 1 patient had an excellent result for four years and then developed recurrent pain and had another arthroscopic shoulder procedure of unknown type elsewhere seven years after their SLAP repair.

## 6 DISCUSSION

Overall good results were seen in this series in all patients who required surgery for their Type 2 SLAP tear by repairing the biceps anchor anatomically without long head of the biceps sacrifice by tenodesis or tenotomy. Regarding patient age, some prior studies have reported worse outcomes in older patients [5, 8], including Denard [9] (although not to statistical significance). However, in our series, older patients did not fare worse than younger patients; and in fact older patients showed greater reductions in pain scores than younger patients. We believe our study is the first to show no difference in results between older and younger patients with superior labral repair. Regarding range of motion, all patients achieved satisfactory motion with a conservative rehabilitation program, with none requiring repeat operation or manipulation under anesthesia.

We believe the trend toward biceps tenodesis or tenotomy among orthopaedic surgeons is at least partially driven by the simplicity of performing the procedure, much as with acromioplasty from prior years. In many cases, the biceps is pristine without damage, in patients who nonetheless have biceps tenodesis or tenotomy performed on them. We think it is unlikely that sacrificing a healthy structure in this fashion is often indicated.

While short term studies have shown sacrifice of the long head of the biceps to be well tolerated, there is evidence [18] that sacrifice of the long head of the biceps with the short head intact can lead to an increased risk of shoulder impingement. There is also evidence [19] that a supraspinatus deficient shoulder will have increased stress and degeneration of the infraspinatus and subscapularis as well as the articular cartilage if the biceps is sacrificed. Thus the many patients in whom the biceps has been sacrificed in recent years may not manifest deleterious effects until many years in the future, if they develop rotator cuff pathology. There is little if any data as to how they might fare over the long term.

In addition to not sacrificing the biceps tendon when it is undamaged, we also do not sacrifice the biceps tendon even if it is damaged. We do not believe that sacrifice of a damaged biceps tendon is indicated any more than sacrifice of a damaged Achilles or patellar tendon would be: although the biomechanical role of the long head of the biceps is obviously not as clear cut as that of these two tendons Tendons, including the long head of the biceps tendon and unlike hyaline cartilage, have the capacity to heal, and also to perform useful painless function even with some wear and tear – much like almost every other structure in the body. We believe that cutting a functioning biceps tendon is unlikely to stand the test of time as a definitive treatment for SLAP lesions. We believe that anatomic restoration, as demonstrated in this series, is safe and effective. Furthermore, most patients intuitively are not happy with the concept of biceps sacrifice. In our experience they uniformly do not like the idea of one of their tendons being cut, but succumb to it because their surgeon tells them that it is the best road to improvement. And there is increasing evidence that orthobiologic treatment can help even damaged tendons heal [20-22], providing yet another reason to avoid biceps sacrifice.

## 7 STUDY LIMITATIONS

Because surgical indications were conservative, the study cohorts were relatively small: Albeit large enough to validate the proposed study hypotheses. Loss of some SST data occurred, but the remaining data we believe is adequate to further validate the study hypotheses.

## CONCLUSION

Repair of the biceps anchor is a successful treatment for type 2 SLAP lesions and does not increase the risk of stiffness. At surgery, it is important to not tighten the biceps or superior labrum beyond their anatomic state. Superior labral biceps anchor repair is equally as effective for older patients as for younger patients who require surgery: without biceps tenotomy or tenodesis.

## Figures and Tables

**Table 1:** Follow-up results – totals and by age sub-group.

–	–	–	**All Patients**	**Age 39 & Less**	**Age 40 & Over**	**P Value Between Age Groups**
**SANE** (0-100, 0 No Pain)	Pre OP	#	20	11	9	
		Mean	5.25	39.5	68.3	p = 0.01
		St Dev	27	26.5	18.4	
	Post Op	#	24	13	11	
		Mean	13.5	10.2	17.5	p = 0.51
		St Dev	22.7	18.2	27.6	
	p value before/after		p < 0.01	p=0.01	p < 0.01	
**Simple Shoulder Test (SST) -** (0-12, 12 best score)	Pre OP	#	22	12	10	
		Mean	7.8	8.5	6.9	p = 0.17
		St Dev	2.7	3.0	2.1	
	Post Op	#	24	13	11	
		Mean	11.3	11.6	10.8	p = 0.38
		St Dev	2.1	0.7	3.1	
	p value before/after		p < 0.01	p < 0.01	p < 0.01	
**Patient Satisfaction**	#		22	11	11	
	Definitely would do surg again		13	5	8	
	Probably would do surg again		8	5	3	
	Probably would not do surg again		1	1	0	
	Definitely would not do surg again		0	0	0	

**Table 2:** Demographics of Sub-Groups.

–	–	**All Patients**	**Age 39 & Less**	**Age 40 & Over**
**Demographics**	Total Pts	30	18	12
	Male/Female	25/5	14/4	11/1
	Mean Age	31	23.4	50.4
	Mean Mo to FU	57.5	64.1	49.6
	Isolated SLAP	20	12	8
	SLAP w/DC	4	2	2
	SLAP w/microfx	2	1	1
	SLAP w/loose body	1	1	0
	SLAP w/Bankart	3	2	1

## LIST OF ABBREVIATIONS

AROM: Active Range of Motion
FF: Forward Flexion
SANE: Single Assessment Numeric Evaluation
SLAP: Superior Labrum Anterior Posterior
SST: Simple Shoulder Test
